# Tacrolimus Dose Requirement in *De Novo* Adult Kidney Transplant Patients Treated With Adoport^®^ Can Be Anticipated

**DOI:** 10.3389/ti.2024.13495

**Published:** 2024-10-14

**Authors:** Pierre Marquet, Dany Anglicheau, Antoine Humeau, Sofian Adrouche, Lakhdar Saada, Julie Bisiaux, Sara Guillemin, Audrey Lardy-Cléaud, Lionel Rostaing

**Affiliations:** ^1^ Department of Pharmacology, Toxicology and Pharmacovigilance, Centre Hospitalier Universitaire de Limoges, Limoges, France; ^2^ Pharmacology and Transplantation, UMR1248 Inserm Université de Limoges, Limoges, France; ^3^ Department of Nephrology and Kidney Transplantation, Necker Hospital, Université Paris Cité, Paris, France; ^4^ Medical Department, SANDOZ S.A.S, Levallois-Perret, France; ^5^ Clinical Operations Department, RCTs, Lyon, France; ^6^ Medical Writing Department, RCTs, Lyon, France; ^7^ Statistical Department, RCTs, Lyon, France; ^8^ Department of Nephrology, Centre Hospitalier Universitaire de Grenoble, Grenoble, France

**Keywords:** tacrolimus, pharmacokinetic variability, diet, ethnicity, genetic polymorphisms

## Abstract

All the factors potentially influencing tacrolimus dose requirement and combinations thereof have never been thoroughly investigated, precluding accurate prediction of tacrolimus starting dose. This prospective, non-interventional, multicenter study in *de novo* adult kidney transplant recipients over the first year after transplantation aimed to investigate the factors influencing tacrolimus dose-standardized trough blood concentration (C_0_/D) over the first week post-transplant (D4-D7, primary objective), D8-M3 and M3-M12 (secondary objectives). Statistical analysis employed mixed linear models with repeated measures. Eighteen sites enrolled 440 patients and followed them up for 9.5 ± 4.1 months. Age at baseline (*p* = 0.0144), end-stage renal disease (*p* = 0.0092), CYP3A phenotype (*p* < 0.0001), dyslipidemia at baseline (*p* = 0.0031), hematocrit (*p* = 0.0026), total bilirubin (*p* = 0.0261) and plasma creatinine (*p* = 0.0484) independently increased with log(C_0_/D) over D4-D7, explaining together 72.3% of the interindividual variability, and representing a robust model to estimate tacrolimus initial dose. Donor age and CYP3A phenotype were also influential over D8-M3 and M3-12, in addition to recipient age. Corticosteroids, diabetes at baseline, and ASAT yielded inconstant results between D8-M3 and M3-M12. We found no ethnicity effect when CYP3A phenotype was accounted for, and no food effect. Intra-individual variability over M3-M12 was moderate, and significantly lower in patients with chronic hepatic disorder (*p* = 0.0196) or cancer (*p* = 0.0132).

## Graphical Abstract

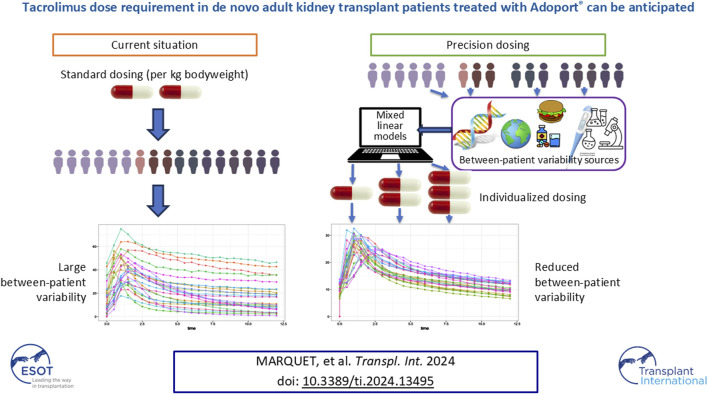

## Introduction

Tacrolimus is an immunosuppressant widely used for the prevention of allograft rejection in solid organ transplantation. However, it is characterized by a narrow therapeutic window and extensive Inter-Individual Variability (IIV) resulting in a challenging determination of the appropriate dose that is both safe and effective at the individual level [1–4]. Rapid achievement of trough blood levels in the desired target range is critical to optimize safety and efficacy during the early post-transplant period [1].

Several clinical studies aimed to identify the variability factors influencing tacrolimus exposure, with the goal of tailoring tacrolimus dose to each patient. The factors known to influence tacrolimus pharmacokinetics include: food-drug interactions, drug-drug interactions and erratic gastrointestinal motility, which all impact tacrolimus absorption velocity or intensity in the gastrointestinal tract; the efflux-pump P-glycoprotein (P-gp) activity, which affects tacrolimus transport back to the digestive lumen; weight and hematocrit that influence tacrolimus distribution; genetic polymorphisms in cytochrome P450 family three subfamily A (CYP3A) enzymes that modulate tacrolimus metabolism and elimination [1, 3, 4].

Ethnicity has also been reported to be a variability factor. In particular, “African-American” transplant patients require higher tacrolimus doses than “Caucasians” to maintain comparable tacrolimus trough concentrations [5–7]. These differences partly arise from variations in intestinal CYP3A or P-gp activities between ethnic groups [8–10]. Dietary habits, which differ between ethnicities, might play a role as well [11].

However, none of these clinical studies extensively evaluated many factors at the same time, including food effect (encompassing the timing of tacrolimus intake and high-fat food consumption), genetic polymorphisms and ethnicity (not only “African-Americans” and “Caucasians”, but in general).

The aim of this study was to thoroughly investigate the different factors influencing tacrolimus exposure over the first week and the first year post-transplant in kidney transplant recipients.

## Patients and Methods

### Study Design and Conduct

This is a prospective, non-interventional, multicenter study conducted at 18 kidney transplant centers in France between April 2018 and October 2020. All the French kidney transplant centers known for using tacrolimus Adoport^®^ (SANDOZ, Levallois-Perret, France) as maintenance therapy for kidney transplant recipients were considered during the site selection process, in order to obtain the most representative population sample.

The primary objective of the study was to investigate the variability factors affecting tacrolimus dose-standardized trough blood concentration (C_0_/D) in adult kidney transplant recipients over the first week post-transplant. Secondary objectives were to investigate the same variability factors and evaluate tacrolimus safety during the first year post-transplant.

The study protocol and its amendments were approved by an Ethics Committee in France (reference 2-17-47, ID-RCB: 2017-A02512-51) and the study was conducted in accordance with the Good Clinical Practice guidelines, the Declaration of Helsinki, the Declaration of Istanbul and all other applicable regulatory requirements. All the participants provided written informed consent.

It is a non-interventional study, meaning that no specific procedures were required as per the protocol. In particular, the investigating sites were already using Adoport^®^ before they were selected for this study and the decision to initiate tacrolimus treatment had been made by their physician before the patients consented to participate in the study. Treatments, patient follow-up schedule, laboratory tests and data collection were performed according to usual clinical practice. Of note, the Summary of Product Characteristics recommends taking Adoport^®^ twice daily [12]. Patients were only asked to fill in questionnaires related to the tacrolimus intake recommendations they received (and remembered) as well as to the timing of tacrolimus intake and what they usually ate for dinner. Data referring to the following timepoints were collected: day of transplant (D0), 7 days post-transplant (D7) or - at the latest - on the day of hospital discharge (baseline), 3 months post-transplant (M3), and 12 months post-transplant (M12) or premature discontinuation; this schedule was chosen to mirror usual clinical care across investigating sites. Tacrolimus whole-blood C_0_ measurements were performed at each site, whereas those to characterize genetic polymorphisms were performed by a central laboratory.

### Patient Population

To minimize selection bias, patients were consecutively included by each site.

Adult recipients of a first kidney allograft, treated *de novo* with tacrolimus (Adoport^®^) for transplant rejection prophylaxis, and for whom the first dose of tacrolimus was taken on the transplantation day or within 24 h post-transplant, were included in the study. The criteria related to first transplant and *de novo* tacrolimus treatment enabled avoiding any previous tacrolimus impregnation. Moreover, the criterion related to tacrolimus initiation on the transplantation day or within 24 h post-transplant ensured timing homogeneity between subjects.

Patients who had a combined transplant, who were taking during the first week post-transplant major enzymatic inhibitors (i.e., azole anti-fungal drugs, protease inhibitors against the human immunodeficiency or the hepatitis C viruses, erythromycin) or major enzymatic inducers (i.e., phenytoin, rifampicin, St John’s Wort) – all known to interact with tacrolimus [12] - or who were participating in an interventional study, were excluded.

### Sample Size

Assuming a correlation rhô = 0.2 between C_0_/D and quantitative factors, and using multiple regression with an alpha risk of 5% and a power of 80%, 400 patients were required to select 10 factors out of the predefined 45 [13]. As it was anticipated that 10% of the patients would not be evaluable (i.e., patients dropping out of the study before M12), at least 440 patients were to be included in the study.

### Statistical Analysis

Analyses were performed on log(C_0_/D) to account for non-normality.

The primary endpoint, which relates to IIV of log(C_0_/D) between D4 and D7 and associated variability factors, was evaluated in all the included patients who met all the eligibility criteria and for whom at least one C_0_/D value was available over D4-D7 [Full Analysis Set (FAS)].

IIV and variability factors of log(C_0_/D) between D8 and M3 as well as between M3 and M12 were analyzed as secondary endpoints in all the included patients who met all the eligibility criteria and for whom at least one C_0_/D value was available over the respective periods [Full Analysis Set 2 (FAS2)].

For the primary objective, we investigated: demographic characteristics and medical history/comorbidities at baseline; the CYP3A phenotype inferred from the CYP3A5*1, *3, *6, *7 and the CYP3A4*22 SNPs, as proposed by Elens et al. [14, 15]; the P-gp phenotype derived from the ABCB1 exons 12, 21 and 26 as proposed by Woillard et al. [16]; transplant characteristics; as well as, over the D0-D7 period, tacrolimus initial dose and number of C_0_/D measurements, concomitant treatments with known interactions with tacrolimus, renal function, laboratory test results, diarrhea and New Onset Diabetes After Transplant (NODAT). For the secondary objectives, we investigated the same variables over the D8-M3 and M3-M12 period, as well as: the existence of a therapeutic education program; the recommendations received at hospital discharge regarding tacrolimus intake in accordance with the physician’s opinion regarding the patient’s understanding of tacrolimus prescription and recommendations at M3 or M12; whether a biopsy was performed before M3; dietary habits and timing of tacrolimus intake as reported by the patient on the questionnaires filled-in at M3 and M12.

It should be noted that the timing of tacrolimus intake over D4-D7 was not evaluated as part of the primary objective because, during this period, patients are still hospitalized, and tacrolimus is presumably administered on an empty stomach by healthcare professionals. CYP3A phenotype was classified as slow, intermediate or rapid based on *CYP3A4* and *CYP3A5* genotypes, as described in Table 1 and following Elens and Haufroid, and Lloberas et al. [14, 15]. P-gp phenotype was classified as slow, intermediate or rapid based on *ABCB1* genotype (slow for homozygous TTT haplotype, intermediate for heterozygous TTT haplotype and rapid for lack of TTT haplotype), following Woillard et al. [16].

**TABLE 1:** Classification of CYP3A phenotypes inferred from the most frequent CYP3A4 and CYP3A5 SNPs following Elens et al. [14].

CYP3A phenotype	*CYP3A5* genotype	*CYP3A4* genotype
Poor	No CYP3A5*1 allele	Heterozygous or homozygous for CYP3A4*22
Intermediate	Homozygous or heterozygous for CYP3A5*1	Heterozygous or homozygous for CYP3A4*22
	No CYP3A5*1 allele	No CYP3A4*22 allele
Extensive	Homozygous or heterozygous for CYP3A5*1	No CYP3A4*22 allele

To investigate the univariate effect of factors, we employed ANOVA or *t*-test for categorial factors, and Spearman rank-order correlation coefficient [with its 95% Confidence Interval (CI_95_)] calculated with the Fisher’s z transformation for continuous factors. Multivariate analyses were run on all the factors with *p* < 0.05 at the univariate tests. If the CYP3A or P-gp phenotypes were significant, they were selected for multivariate analysis and the corresponding genotypes were not. These factors were entered, as fixed effects, in a mixed model for repeated measurements. For all the mixed linear models, the subject was considered as a random effect and the number of days since transplant as a fixed effect. For the IIV the same mixed linear model and parameters, without factors, were used. Intra-individual, or Inter-Occasion Variability (IOV), was studied in patients with at least 3 C0 values available in the eCRF over the M3-M12 period. The influence of the same factors on log(C0) IOV was analyzed using univariate and then multivariate multilinear models.

Tacrolimus safety was evaluated in all the included patients who met all the eligibility criteria and received at least one dose of tacrolimus [Safety Analysis Set (SAS)]. Adverse events were coded using the international Medical Dictionary for Regulatory Activities (MedDRA) reference dictionary version 20.1.

Missing data were not replaced. Sample size calculation was performed using SAS software v9.4, and univariate and multivariate statistical analyses using R v4.4.0 (R Foundation) and the R packages lme4 (v1.1-35.3) and lmerTest v3.1-3.

## Results

### Patient Population

**FIGURE 1:**
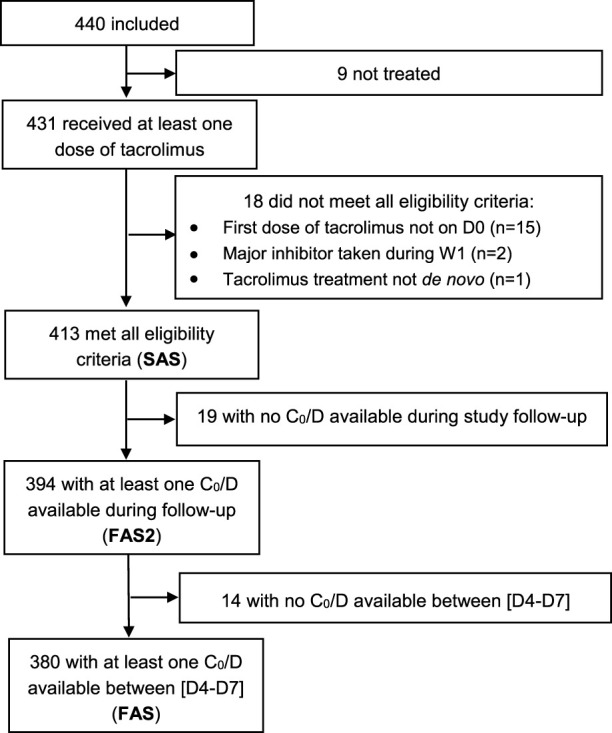
Flowchart of the different analysis populations.

**FIGURE 2:**
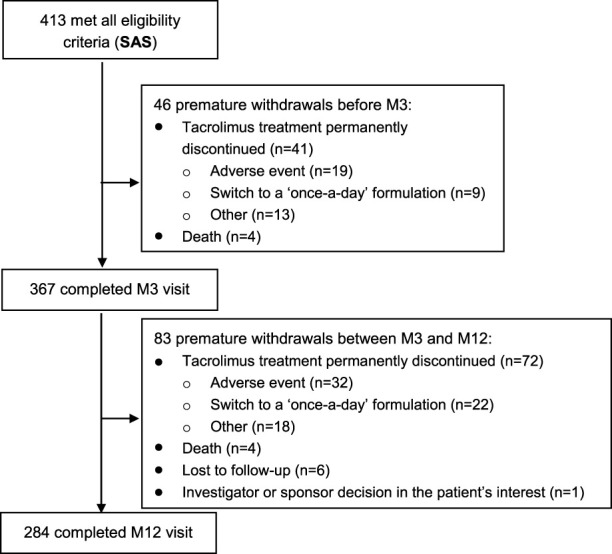
Flowchart of patient follow-up.

In total, 440 patients were included at 18 investigating sites and followed up over 9.5 ± 4.1 months on average. As illustrated in Figure 1, 413 patients constituted the SAS, 394 the FAS2 and 380 the FAS. Regarding follow-up, among the 413 patients of the SAS, 367 (88.9%) completed the M3 visit and 284 (68.8%) completed the M12 visit (Figure 2).

Baseline characteristics of the SAS patients are described in Table 2. Overall, they were aged 55.2 ± 15.2 years [mean ± Standard Deviation (SD)] and 65.1% of them were males. Their mean Body Mass Index (BMI) ± SD was 25.6 ± 4.3 kg/m^2^. Regarding ethnicity, 77.7% of the patients considered themselves as White European, 11.1% as North African or from the Middle East, 9.0% as Black African or Black Caribbean, 1.9% as Asian and 0.2% as Other. These baseline characteristics were similar in FAS and FAS2. At inclusion, 95.7% patients had received an induction treatment and all received immediate-release tacrolimus Adoport^®^, co-administered with a mycophenolate drug (95.9%) and corticosteroids (98.3%).

**TABLE 2:** Baseline characteristics (SAS, n = 413).

Age, mean (SD) (years)	55.2 (15.2)
Male sex, n (%)	269 (65.1)
Height, mean (SD) (cm)	169.8 (10.3)
Weight at D0, mean (SD) (kg)	73.9 (15.1)
BMI, mean (SD) (kg/m^2^)	25.6 (4.3)
Ethnicity according to the patient, n (%)	
White European	321 (77.7)
North African or Middle East	46 (11.1)
Black African or black Caribbean	37 (9.0)
Asian	8 (1.9)
Other	1 (0.2)
Diabetes, n (%)	94 (22.8)
Heart failure, n (%)	19 (24.7)
CYP3A phenotype, n (%)	
Poor	27 (7.1)
Intermediate	253 (66.9)
Extensive	98 (25.9)
Missing	35
P-gp phenotype, n (%)	
Slow	51 (13.5)
Intermediate	150 (39.7)
Rapid	177 (46.8)
Missing	35
Induction treatment, n (%)	
Non-depleting antibodies	219 (53.0%)
Depleting antibodies	175 (42.4%)
None except corticosteroid bolus	19 (4.6%)
Adoport^®^ starting dose, mean (SD) (mg)	7.1 (3.5)
Other maintenance immunosuppressive treatment at inclusion	
Mycophenolate mofetil or mycophenolic acid sodium salt, n (%)	396 (95.9%)
Corticosteroids, n (%)	406 (98.3%)

### Variability of Tacrolimus log(C_0_/D) Over D4-D7

The IIV of log(C_0_/D) over D4-D7 was 0.32 (CI_95_ [0.28; 0.38]). Univariate analysis results are presented in Supplementary Table S1. Among the 21 factors considered in the multivariate model, those that influenced tacrolimus log (C_0_/D) in the FAS were: recipient age at baseline (*p* = 0.0144), the main cause of end-stage renal disease (*p* = 0.0092); CYP3A phenotype (*p* = 0.0001); dyslipidemia at baseline (*p* = 0.0031); and hematocrit (*p* = 0.0026), total bilirubin (*p* = 0.0261) and plasma creatinine (*p* = 0.0484) over the D4-D7 period (Table 3). Together, they explain 72.3% of the IIV, while IOV explained another 19.2%.

**TABLE 3:** Multivariate analysis of potential variability factors of tacrolimus log(C_0_/D) over D4-D7 (FAS, n = 380), D8-M3 (FAS2, n = 394) and M3-M12 (FAS2, n = 394).

Variable	D4-D7 (primary objective)		D8-M3		M3-M12	
	Beta	*P*-value	Beta	*P*-value	Beta	*P*-value
Donor age	−0.0042 [−0.0162; 0.0078]	0.4976	−0.0084 [−0.0149; −0.0019]	**0.0121**	−0.0081 [−0.0148; −0.0014]	**0.0190**
Recipient age at baseline	0.0193 [0.0043; 0.0343]	**0.0144**	0.0087 [0.0012; 0.0162]	**0.0245**	0.0104 [0.0027; 0.0180]	**0.0083**
Recipient gender (vs. male)	0.1998 [−0.0356; 0.4351]	0.1008				
Ethnicity (vs. White European, n = 302*)						
Asians (6)	−0.0358 [−0.8076; 0.7360]	0.1702	0.6749 [0.1620; 1.1879]	0.0951	0.5527 [0.0386; 1.0668]	0.1437
Black Africans and Caribbeans (34)	0.6975 [0.0388; 1.3561]		−0.0121 [−0.2577; 0.2335]		0.2448 [0.0052; 0.4845]	
North Africans and Middle East (37)	0.0787 [−0.3809; 0.5382]		−0.0691 [−0.2883; 0.1500]		0.1039 [−0.1170; 0.3248]	
Others (1)	NA	NA	0.2167 [−0.5698; 1.0032]		0.1275 [−0.7407; 0.9957]	
CYP3A phenotype: intermediate vs. rapid	0.7814 [0.4042; 1.1585]	**0.0001**	0.6662 [0.4996; 0.8328]	**<10** ^**−**^ **^14^**	0.8696 [0.6962; 1.0430]	**<10** ^**−**^ **^20^**
CYP3A phenotype: slow vs. rapid	1.5607 [0.8755; 2.2459]		0.9810 [0.7293; 1.2327]		1.2112 [0.9444; 1.4780]	
P-gp phenotype: intermediate vs. rapid	−0.0037 [−0.2715; 0.2642]	0.6829	−0.0412 [−0.1690; 0.0866]	0.5590		
P-gp phenotype: slow vs. rapid	−0.1281 [−0.4441; 0.1880]		0.0595 [−0.1334; 0.2524]			
Main cause of end-stage renal disease (vs. hypertension, n = 62)						
Chronic interstitial nephropathy and pyelonephritis (24)	−0.4915 [−0.9719; −0.0111]	**0.0092**	−0.1584 [−0.4415; 0.1246]	0.4863		
Diabetes mellitus (39)	0.1143 [−0.3641; 0.5926]		−0.0713 [−0.3320; 0.1894]			
Dysimmune nephropathy including lupus and vascularitis (11)	−0.9491 [−1.7282; −0.1699]		−0.0293 [−0.3896; 0.3310]			
Glomerulopathy including IgA nephropathy (90)	0.4351 [0.0297; 0.8406]		−0.1901 [−0.3935; 0.0132]			
Polycystic kidney disease (73)	0.0880 [−0.2981; 0.4741]		−0.1547 [−0.3513; 0.0419]			
Uropathy including reflux nephropathy (13)	−0.1868 [−0.7753; 0.4016]		0.0355 [−0.3174; 0.3883]			
Undetermined (68)	−0.1384 [−0.5187; 0.2420]		−0.2046 [−0.4012; −0.0080]			
Other (14)	0.5866 [−0.0173; 1.1905]		−0.3229 [−0.7408; 0.0949]			
Cardiovascular disease (Y/N)	0.2285 [−0.1214; 0.5783]	0.2052	0.1528 [−0.0047; 0.3103]	0.0587		
Diabetes at baseline (Y/N)	0.2029 [−0.1981; 0.6039]	0.3253	0.0932 [−0.1025; 0.2889]	0.3517	0.1639 [0.0142; 0.3135]	**0.0328**
Dyslipidemia at baseline (Y/N)	0.3757 [0.1370; 0.6145]	**0.0031**	0.0571 [−0.0706; 0.1847]	0.3820	0.0379 [−0.0880; 0.1639]	0.5555
BMI at D0	−0.0002 [−0.0307; 0.0302]	0.9895				
Requirement for dialysis over the 1st week post-transplant (Y/N)	−0.1052 [−0.5217; 0.3113]	0.6223				
Number of dialyses	0.0105 [−0.0741; 0.0951]	0.8081				
Corticosteroids (Y/N)	NA	NA	−0.1501 [−0.2972; −0.0029]	**0.0470**		
Laboratory test results over the targeted periods						
Total bilirubin	0.0517 [0.0071; 0.0964]	**0.0261**	0.0001 [−0.0080; 0.0083]	0.9756		
ASAT					0.0034 [0.0012; 0.0056]	**0.0023**
Gamma GT	−0.0003 [−0.0013; 0.0007]	0.5840			0.0002 [−0.0002; 0.0005]	0.4329
Hematocrit	0.0398 [0.0147; 0.0649]	**0.0026**				
Plasma creatinine	0.0009 [0.0000; 0.0018]	**0.0484**				
eGFR	0.0006 [−0.0033; 0.0045]	0.7606				
* Urine creatinine*	−0.0189 [−0.0500; 0.0122]	0.2394				
Number of C_0_/dose measurements	0.0596 [−0.0567; 0.1759]	0.3187				
Timing of tacrolimus intake (during meals vs. outside meals)	NA	NA	−0.0741 [−0.2978; 0.1497]	0.5172		

*Patient numbers in brackets are for the period D0-D7.

NA, not assessed.

Cells are left empty when variables were not significant at the univariate stage.

Significant p-values (<0.05) are in bold characters.

### Variability of Tacrolimus log(C_0_/D) Over D8-M3

The IIV of log(C_0_/D) over D8-M3 was 0.27 (CI_95_ [0.23; 0.31]). Among the 12 factors (including “timing of tacrolimus intake”) selected for the multivariate model, those that affected tacrolimus log (C_0_/D) in the FAS2 were: donor age (*p* = 0.0121), recipient age at baseline (*p* = 0.0245), CYP3A4 slow or intermediate phenotype (*p* < 10^−14^) and corticosteroid treatment over the time period (*p* = 0.0470), explaining 33.5% of the total variability. The IOV explained another 40.4%.

### Variability of Tacrolimus log(C_0_/D) Over M3-M12

The IIV of log(C_0_/D) over M3-M12 was 0.33 (CI_95_ [0.28; 0.39]). Among the eight factors selected for the multivariate model, those that affected tacrolimus log (C_0_/D) in the FAS2 were: donor age (*p* = 0.0190), recipient age at baseline (*p* = 0.0083), CYP3A4 slow or intermediate phenotype (*p* < 10^−20^), diabetes at baseline (*p* = 0.0328) and ASAT over the time period (*p* = 0.0023), explaining 36.4% of the total variability (as compared with 46.9% for the IOV).

In 254 patients with at least 3 C0 values saved in the eCRF over the M3-M12 period, IOV (intra-individual variability) of log(C_0_/D) and C_0_/D over the same time period was moderate (Table 4). However, there were a few outliers with CV > 40%, up to 92.2% (Supplementary Figure S2). The IOV was significantly lower in patients with chronic hepatic disorder or cancer (Supplementary Table S3).

**TABLE 4:** Intra-individual variability (IOV) of log(C_0_/dose) and C_0_/dose over the M3-M12 period.

Metrics	IOV of (C_0_/dose)	IOV of log (C_0_/dose)
Minimum	0	−23.292
1st quartile	0.149	−0.234
Median	0.212	0.274
Mean	0.241	−0.023
3rd quartile	0.283	0.568
Maximum	0.922	17.857

### Safety

Out of the 413 patients in the SAS, 369 (89.3%) experienced at least one Treatment-Emergent Adverse Event (TEAE) (i.e., adverse events that occurred after the first tacrolimus intake), for a total of 2,477 TEAEs. TEAEs reported in more than 5% of the patients are presented in Table 5. The most common were anemia (26.9% of the patients, 120 TEAEs), diarrhea (22.8%, 117 TEAEs), tremor (17.2%, 72 TEAEs), hypertension (15.3%, 67 TEAEs) and leukopenia (13.8%, 77 TEAEs). Of note, transplant rejection occurred in 5.1% of the patients (21 TEAEs). Out of these 2,477 TEAEs, 377 (that occurred in 190 patients (46.0%)) were deemed to have a suspected causality to tacrolimus by the investigators.

**TABLE 5:** TEAEs.

MedDRA System Organ Class - Preferred Term	Number of TEAEs	Number of patients	Percentage of patients
**Any TEAEs**	**2,477**	**369**	**89.3**
**Blood and lymphatic system disorders**	**336**	**194**	**47.0**
- Anemia	120	111	26.9
- Leukopenia	77	57	13.8
- Neutropenia	50	43	10.4
- Thrombocytopenia	25	22	5.3
**Gastrointestinal disorders**	**235**	**148**	**35.8**
- Diarrhea	117	94	22.8
**General disorders and administration site conditions**	**122**	**90**	**21.8**
- Oedema peripheral	48	41	9.9
**Hepatobiliary disorders**	**48**	**40**	**9.7**
- Hepatocellular injury	23	22	5.3
**Immune system disorders**	**36**	**32**	**7.7**
- Transplant rejection	21	21	5.1
**Infections and infestations**	**291**	**182**	**44.1**
- BK virus infection	29	29	7.0
- Cytomegalovirus infection	41	38	9.2
- Urinary tract infection	38	34	8.2
**Injury, poisoning and procedural complications**	**146**	**106**	**25.7**
- Delayed graft function	23	23	5.6
- Overdose	31	25	6.1
**Metabolism and nutrition disorders**	**369**	**167**	**40.4**
- Diabetes mellitus	39	39	9.4
- Hyperkaliemia	34	31	7.5
- Hypokalemia	40	31	7,5
- Hypophosphatemia	37	33	8.0
- Metabolic acidosis	25	23	5.6
**Nervous system disorders**	**116**	**98**	**23.7**
- Tremor	72	71	17.2
**Renal and urinary disorders**	**244**	**150**	**36.3**
- Acute kidney injury	69	48	11.6
- Renal impairment	38	35	8.5
**Respiratory, thoracic and mediastinal disorders**	**59**	**50**	**12.1**
- Dyspnea	21	21	5.1
**Vascular disorders**	**144**	**111**	**26.9**
- Hypertension	67	63	15.3
- Lymphocele	22	21	5.1

Among the 2,477 TEAEs, 703 [which occurred in 238 patients (57.6%)], were considered as serious TEAEs. The most common ones were acute kidney injury (9.4% of the patients, 56 TEAEs) and anemia (7.5%, 34 TEAEs). Ninety-two (92) serious TEAEs [that occurred in 74 patients (17.9%)] were deemed to have a suspected causality to tacrolimus by investigators.

Nine patients (2.2%) died during the study following the occurrence of one or several TEAEs, none of which had a suspected causality to tacrolimus.

## Discussion

To the best of our knowledge, this study is the first to investigate so extensively the combination of variability factors influencing tacrolimus dose-standardized exposure (also called ‘dose requirement’), including food effect (through both timing of tacrolimus intake and high-fat food consumption), CYP3A and P-gp phenotypes, and ethnicity (as mostly represented in France). In their comprehensive review article, Vanhove et al. presented evidence for each of them separately, but not combined [17]. Tacrolimus oral clearance is known to decrease progressively over the first 3–9 months post-transplant [18], explaining a natural decrease in C_0_/dose which, together with a decreasing risk of rejection, justifies progressive lowering of tacrolimus doses over the first year post-transplant. For this reason, we considered three time periods in this study, from the first days after surgery up to 1 year.

Multivariate analysis evidenced seven factors significantly influencing tacrolimus log(C_0_/D) over D4-D7 (primary endpoint): recipient age at transplantation, the main cause of end-stage renal disease, CYP3A phenotype (encompassing the CYP3A5 *1, *3, *6, *7 the CYP3A4*22 and the POR*28 SNPs to account for ethnic diversity), dyslipidaemia at baseline and hematocrit, total bilirubin and plasma creatinine over the time period. Together, these seven factors explain 72.3% of C_0_/D variability, meaning that they may be leveraged to adapt the initial dose of tacrolimus to each patient, probably more effectively than previous attempts limited to the CYP3A5*3 genotype [19, 20].

Multivariate analysis of the same variables plus the timing of tacrolimus intake over the other two time periods (D8-M3 and M3-M12) only confirmed the steady influence of recipient age at baseline and CYP3A phenotype. Donor age only reached significance at these later two periods. In contrast, the main cause of end-stage renal disease lost significance after D7. The other variables (dyslipidemia at baseline, diabetes at baseline, corticosteroid treatment, ASAT, haematocrit, plasma creatinine) were only significant at one period.

Regarding CYP3A, the slow/intermediate metabolizing phenotype was associated with higher log (C_0_/D), as expected [4, 14, 15, 21]. Indeed, when CYP3A activity is slow or intermediate, tacrolimus is less metabolized [4], which decreases dose requirement.

The significant influence of recipient age at baseline on tacrolimus dose-standardized exposure in adults may be related to decreased absorption rate [22] and/or increased volume of distribution due to changing body composition with age. Potential confounders of this age effect, such as concomitant treatments, particularly with CYP34 inhibitors or inducers, did not pass univariate analysis. Corticosteroids are known to affect the oral bioavailability of tacrolimus [23] through P-gp and CYP3A4 induction [24]. An observational study in 83 renal transplant recipients showed that the higher the steroid dosage, the higher the dosage of tacrolimus needed to achieve target trough levels in these patients. The most likely interaction mechanism is specific enzymatic induction of CYP3A and/or P-gp and this interaction is present, even when the steroid dosage is low [25]. With regards to dyslipidemia, the SmPCs of several tacrolimus formulations list hyperlipidemia, hypercholesterolemia and hypertriglyceridemia as frequent adverse effects. In addition, significant associations between Tac C0 and hyperlipidemia were reported by several groups, e.g., in 132 Korean kidney transplant recipients using multivariate analysis [26], or in 63 European kidney transplant patients for hypercholesterolemia and hypertriglyceridemia [27]. However, the causality of this association may go both ways, because *in vitro*, 60% of tritiated ciclosporine or tacrolimus are transported by HDL-cholesterol in normolipemic sera, whereas approx. 50%–60% are transported by LDL-cholesterol in hypertriglycemic sera [28].

Donor age was significant at two different periods, suggesting a “false negative” result at D4-D7 or an interaction with time. However, the underlying mechanism of the influence of donor age on tacrolimus IIV is not obvious, since tacrolimus is not substantially excreted in urine.

The inconstant statistical results across the three time periods may be chance findings, but most of them have already been reported in the literature and have plausible pathophysiological explanations. First of all, the relationship between hematocrit and tacrolimus log(C_0_/D) at D4-D7 was expected since tacrolimus in blood is highly bound to red blood cells [29]. This is also consistent with the literature [30–35]. Secondly, the influence of diabetes at baseline on tacrolimus variability in the latest time period may be due to its influence on the interstitial cells of Cajal, the gastric pacemaker cells [36], resulting in delayed gastric emptying [37] and a flatter pharmacokinetic profile, with lower C_max_ and higher C_0_. This is contrasted with the absence of association with new-onset diabetes, possibly due to a much shorter exposure to diabetes. The association of C_0_/D with dyslipidemia found at D4-D7 was not confirmed at the later time periods, maybe because it is favoured by early post-transplant cholestasis, which disappears rapidly [38, 39]. The (weak) link with plasma creatinine in the same period may be more a consequence than a cause of high C_0_/D.

The absence of statistical association between tacrolimus pharmacokinetic IIV and some variables is also interesting. The P-gp phenotype resulting from the combination of the most influential genotypes [40–43] did not show significant effect on log(C_0_/D), although tacrolimus is a substrate of P-gp, that tends to oppose its digestive absorption and favor the biliary and renal elimination of its metabolites [44]. However, tacrolimus dose in the gut lumen probably saturates P-gp efflux capacities, and tacrolimus is hardly excreted unchanged in bile or urine.

Also, contrary to what was expected, no association between log(C_0_/D) and high-fat food consumption was identified as a result of univariate analysis over D8-M3 and M3-M12 (it was not tested at the first time period since the patients were hospitalized). High-fat meals influence both the rate and intensity of oral tacrolimus absorption [1, 12], which is the reason why the tacrolimus label recommends taking it on an empty stomach, that is, at least 1 h before or 2 h after eating [1]. In the present study, high-fat food consumption was defined as the consumption of at least two types of high-fat food during dinner, at least twice a week. Over D8-M12, no statistical association was observed between log(C0/D) and tacrolimus intake during meals. The causes of this apparent discrepancy with the drug label may be: that the regulator recommends that the food effect is evaluated based on the AUC and Cmax in healthy volunteers receiving a single drug dose [45], as opposed to steady-state C0 in patients here; and that we considered high-fat meals when at least two categories of high-fat food were ingested, which in the absence of quantities may not match the FDA definition. As a reminder, the FDA recommends “a high-fat (approximately 50 percent of total caloric content of the meal) and high-calorie (approximately 800–1,000 calories) meal as a test meal for food-effect bioavailability. This test meal should derive approximately 150, 250, and 500–600 calories from protein, carbohydrate, and fat, respectively” [45].

No association between log(C_0_/D) and ethnicity was identified as a result of multivariate analysis at any time period, probably because ethnicity was confounded by the CYP3A and possibly the P-gp phenotypes [46, 47]. Actually, significant association was found between ethnicity and the CYP3A phenotype (*p* < 0.001) when studying the collinearity or covariation of the potential covariates (Supplementary Figure S1). For this reason, we re-ran the multivariate analyses without considering the CYP3A phenotype (Supplementary Table S2), unveiling a significant influence of ethnicity at all periods (*p* = 0.0234, <10^−4^ and <10^−4^ at D4-D7, D8-M3 and M3-M12, respectively) and confirming confusion between the two groups of variables, but also showing that the models with the CYP3A phenotype account for much more variability than those with ethnicity (72.3% vs. 60.9% at D4-D7, 33.5% vs. 15.9% at D8-M3 and 36.4% vs. 13.1% at M3-M12).

Tacrolimus is most often presented as a drug with large IIV and IOV. This study shows that IOV is actually moderate, from 19.2% over D4-D7 to 24.1% over M3-M12. This is not surprising, since individual dose adjustment would be useless in case of short- or mid-term large IOV. It is most probably larger over the full first year post-transplantation, due to the progressive “maturation” of tacrolimus oral clearance, which is the reason why we focused on the latest period to evaluate the determinants of IOV. Despite being moderate on average, IOV was much larger (ca. between 40% and 92%) in a minority of patients, and many studies showed that it is an independent risk of treatment failure [48]. In contrast, IOV was lower in patients with chronic hepatic disorder or cancer. Hepatic disorders may result in a lower metabolic capacity, but the link with a lower IOV is not straightforward. We have no explanation to offer either for the impact of cancer on IOV. Both findings should obviously be confirmed in independent patient populations. Another interesting finding is that none of the other factors, including CYP3A or P-gp phenotypes, ethnicity and food effect, had a significant influence on IOV.

Regarding safety, a high number of TEAEs was expected in view of patient conditions and polypharmacy. Indeed, the primary kidney disease, existing comorbidities, surgery itself, and numerous concomitant therapies may result in many adverse events in the early post-transplant phase [12, 49]. The high number of TEAEs with a suspected causality to tacrolimus was also expected, and is in line with tacrolimus safety profile in adult recipients of a first kidney allograft [50]. All reported TEAEs were known and there was no unexpected safety signal.

This study presents several limitations. First, only a few patients declared themselves as Asians (1.9%), resulting in limited representativity of this group. Moreover, Asia is made up of multiple ethnicities with wide variations in the frequency of CYP3A and ABCB1 polymorphisms between them, and for this reason we recommend replicating this study in Asia [46]. More generally, the present results might not be extrapolated to people from origins not or poorly represented in the study. Also, considering that we only enrolled *de novo* adult kidney transplant patients, our results might not be extrapolated to pediatric patients, or patients transplanted with another organ.

In summary, this prospective, non-interventional, multicenter study, conducted in 440 *de novo* adult kidney transplant patients treated with twice daily tacrolimus, evaluated the combined influence of the timing of tacrolimus intake, high-fat food consumption, CYP3A and P-gp phenotypes, ethnicity and many other variability factors on tacrolimus exposure over the first week and up to 1-year post-transplant in a real-life setting. Over D4-D7, recipient age at baseline, the main cause of end-stage renal disease, CYP3A phenotype, dyslipidemia at baseline and hematocrit, total bilirubin and plasma creatinine over the time period influenced tacrolimus exposure. Together with the multivariable model developed, they may be leveraged to determine the initial dose of tacrolimus. Recipient age at baseline and the CYP3A phenotype were also found to be variability factors over D8-M3 and M3-12, whereas the use of corticosteroids, diabetes at baseline, and ASAT yielded inconstant results between D8-M3 and M3-M12. Tacrolimus intake during meals and high-fat food consumption had no significant influence, while ethnicity was confounded by the CYP3A phenotype. Finally, intra-individual variability in the more stable period M3-M12 was mild and was only influenced by hepatic disorder and cancer, not by CYP3A or Pgp phenotypes, nor ethnicity.

## Data Availability

The authors will make the study protocol and statistical analysis plan available upon request to interested researchers. The data cannot be shared for legal, ethical and patient privacy restrictions.

## Abbreviations

ASAT, ASpartate Amino Transferase; BMI, Body Mass Index; CI_95_, 95% Confidence Interval; C_0_/D, dose-standardized trough blood concentration; CYP3A, cytochrome P450 family 3 subfamily A; D0, day of transplant; D7, 7 days post-transplant; D8, 8 days post-transplant; FAS, Full Analysis Set; FAS2, Full Analysis Set 2; IIV, Inter-Individual Variability; IOV, Inter-Occasion Variability; MedDRA, Medical Dictionary for Regulatory Activities; M3, 3 Months post-transplant; M12, 12 Months post-transplant; NODAT, New Onset Diabetes After Transplant; P-gp, P-glycoprotein; SAS, Safety Analysis Set; SD, Standard Deviation; TEAE, Treatment-Emergent Adverse Event.
