# Supplementary material for: Tacrolimus Dose Requirement in De Novo Adult Kidney Transplant Patients Treated With Adoport® Can Be Anticipated
Source: Transpl Int. 2024 Oct 14;37:13495. doi: 10.3389/ti.2024.13495 (PMC11513580; doi:10.3389/ti.2024.13495)
Supplement: Supplementary file 1 [file DataSheet1.PDF]

Tacrolimus dose requirement in de novo adult kidney transplant patients treated with Adoport® can be anticipated

Pierre Marquet,[1,2,*] Dany Anglicheau,[3] Antoine Humeau,[2] Sofian Adrouche,[4] Lakhdar Saada,[4] Julie Bisiaux,[5] Sara Guillemin,[6] Audrey Lardy-Cléaud,[7] Lionel Rostaing[8]

1 Department of pharmacology, toxicology and pharmacovigilance, Centre Hospitalier Universitaire de Limoges, Limoges, France

2 Pharmacology & Transplantation, UMR1248 Inserm Université de Limoges, Limoges, France

3 Department of nephrology and kidney transplantation, Necker Hospital, Université Paris Cité, Paris, France

4 Medical department, SANDOZ S.A.S, Levallois-Perret, France

5 Clinical operations department, RCTs, Lyon, France

6 Medical writing department, RCTs, Lyon, France

7 Statistical department, RCTs, Lyon, France

8 Department of nephrology, Centre Hospitalier Universitaire de Grenoble, Grenoble, France

*Correspondence: Pierre Marquet, MD; Department of pharmacology, toxicology and pharmacovigilance, CHU Limoges, 2 avenue Martin Luther King, 87000 Limoges, France; pierre.marquet@unilim.fr

SUPPLEMENTAL MATERIAL

Table S1: Univariate analysis of variability factors of tacrolimus log(C_0/D) over D4-D7 (FAS, n=380), D8-M3 (FAS2, n= 394) and M3-M12 (FAS2, n= 394).

		p values		
	**Variables tested**	**D4-D7**	**D8-M3**	**M3-M12**
**Patient characteristics**	Ethnicity	**< 0.0001**	**< 0.0001**	**< 0.0001**
	Gender	**0.044**	0.5404	0.7219
	Age at transplantation	**< 0.0001**	**< 0.0001**	**0.0005**
	Height	**0.0001**	**0.0168**	0.3663
	Weight at D0	0.701	-	-
	Weight at D7	-	0.7074	-
	Weight at M3	-	-	0.459
	BMI at D0	**0.042**	-	-
	BMI at D7	-	0.0833	-
	BMI at M3	-	-	0.1065
**Transplantation cause and conditions**	Main cause of end-stage renal disease	**0.001**	**0.0315**	0.3578
	Donor type	0.614	0.4799	0.306
	Age of the donor	**< 0.0001**	**0.0003**	**0.0017**
	Time to resumption of gastrointestinal function	0.96	0.8441	0.1905
	Additional surgery required in the first week post-transplant	0.988	0.9067	0.8794
	Additional surgery required in the first week post-transplant: general anaesthesia	0.887	0.7855	0.1273
	Additional surgery required in the first week post-transplant: digestive or intraperitoneal surgery	0.16	0.8815	0.4974

Tables S1 (continued)

	Variables tested	p values		
		**D4-D7**	**D8-M3**	**M3-M12**
**Medical history/comorbidities**	Arterial hypertension (Y/N)	0.736	0.2224	0.8775
	Arterial hypertension (uncontrolled, controled, none)	0.699	0.6214	0.7676
	Chronic hepatic disorder	0.4	0.6236	0.7511
	Cardiovascular disease	**0.0069**	**0.0452**	0.1342
	Cardiovascular disease /cerebrovascular accident	**0.0259**	0.1197	0.1665
	Cardiovascular disease/ Myocardial infarction	**0.0179**	0.1339	0.226
	Cardiovascular disease/Heart failure	**0.015**	0.1236	0.3129
	Arterial/venous thromboembolic disease	0.709	0.6214	0.663
	Cancer	0.353	0.7811	0.8317
	Dyslipidaemia	**0.0266**	**0.0165**	**0.0289**
	Pre-transplant diabetes	**< 0.0001**	**0.0032**	**0.0101**
	Bariatric surgery (Y/N)	0.667	0.572	0.5069
	Bariatric surgery (category)	0.765	0.1542	0.3427
	Occurrence of diarrhoea over the targeted periods	0.5123	0.1799	0.6019
**Kidney function**	Time to kidney function recovery after tx	0.138	0.9772	0.9332
	Requirement for dialysis over the 1st week post tx	**0.005**	0.2581	0.2307
	Number of dialyses	**0.002**	0.2005	0.4299
	Graft rejection since tx	0.682	0.3407	0.5509
	Plasma creatinine	**0.039**	0.7438	0.9582
	eGFR	**0.0005**	0.0556	0.776
	Proteinuria	0.262	0.8758	0.4236
	Urine creatinine	**0.0002**	0.066	0.9582
**Graft biopsy**	Biopsy performed before M3	-	0.497	0.314
**Post-transplant diabetes**	New-onset diabetes over the period of interest	0.142	0.3895	0.0633
**Laboratory test results over the targeted periods**	Hematocrit	**< 0.0001**	0.0591	0.0801
	ASAT	0.202	0.4673	**0.0321**
	ALAT	0.762	0.7036	0.1
	Gamma GT	**0.024**	0.1633	**0.0081**
	total bilirubin	**0.003**	**0.0009**	0.172

Tables S1 (continued)

	Variables tested	p values D4-D7	p values D8-M3	p values M3-M12
**Adoport dose**	Initial daily dose (mg)	0.532	0.2348	0.4754
	Initial daily dose (mg/kg)	0.528	0.1036	0.069
**Other IS drugs over the targeted periods**	mTOR inhibitor (Y/N)	0.182	0.972	0.5157
	Immunosuppressive induction treatment (Y/N)	0.404	0.0537	0.7516
	Mycophenolate (Y/N)	0.66	0.2418	0.3281
	Corticosteroids (Y/N))	0.937	**0.0396**	0.732
	DDI between tacrolimus and CYP3A inducers or inhibitors (induction+inhibition±neutral / induction±neutral / inhibition±neutral / none)	0.978		
	Introduction or discontinuation of drugs with DDI with tacrolimus at D8-M3 (Y/N)		0.1084	
	Introduction or discontinuation of drugs with DDI with tacrolimus at M3-M12 (Y/N)			0.2291
**Genetic polymorphisms / phenotypes**	CYP3A5*1	-	-	-
	CYP3A5*3	**< 0.001**	**< 0.0001**	**< 0.0001**
	CYP3A5*6	**0.002**	**0.0002**	0.2597
	CYP3A5*7	0.799	0.331	0.9756
	CYP3A4*22	0.363	**0.0008**	**0.0185**
	POR*28	0.764	0.384	0.8549
	ABCB1 exon 12	0.523	0.186	0.7965
	ABCB1 exon 21	0.072	**0.0189**	0.2767
	ABCB1 exon 26	**0.031**	**0.0425**	0.9535
	ABCB1 c.1199G>A	0.947	0.1913	0.4987
	CYP3A phenotype	**< 0.0001**	**< 0.0001**	**< 0.0001**
	P-gp phenotype	**0.007**	**0.0045**	0.1607
	biopsy before M3 results	-	0.8479	0.8928

Tables S1 (continued)

	Variables tested	p values D4-D7	D8-M3	M3-M12
**Food effect**	Recommendations received at discharge regarding timing of tacrolimus intake by the patient (Y/N)	-	0.7347	0.1053
	Physician's opinion at M3 as to whether each patient understands their prescription and drug administration recommendations: number of daily intakes	-	0.9795	0.7139
	Physician's opinion at M3 as to whether each patient understands their prescription and drug administration recommendations: timing of tacrolimus intake	-	0.9669	0.4094
	Timing of tacrolimus intake vis a vis food at M3	-	**0.012**	0.1664
	Timing of tacrolimus intake vis a vis food at M12		**-**	0.5535
	Patient dietary habit in relation to drug administration at M3	-	0.5497	0.4897
	Patient dietary habit in relation to drug administration at M12	-	-	0.3629
**C0/D measurement conditions**	Days since transplantation of C0/D values	0.5925	0.2342	0.661
	Number of C0/D values per patient	**0.0026**	0.8319	0.8217

Table S2: Sensitivity analysis of the confounding effect of CYP3A phenotype on ethnicity. Multivariate analysis of potential variability factors of tacrolimus log(C_0/D) over D4-D7 (FAS, n=380), D8-M3 (FAS2, n=394) and M3-M12 (FAS2, n=394)

	**Period D3-D7 (main objective)**		**Period D8-M3**		**Period >M3-M12**	
**Variable**	**Beta [2.5%; 97.5%]**	**P value**	**Beta**	**P value**	**Beta**	**P value**
Donor age	0.0058 [-0.0066; 0.0182]	0.3634	-0.0023 [-0.0090; 0.0044]	0.5042	0.0016 [-0.0056; 0.0088]	0.6703
Recipient age at baseline	0.0016 [-0.0128; 0.0161]	0.8248	0.0058 [-0.0018; 0.0134]	0.1378	0.0029 [-0.0052; 0.0111]	0.4829
Recipient gender	0.1169 [-0.1415; 0.3753]	0.3781				
Ethnicity (vs. White European, n = 302*)						
Asians (6)	-1.0585 [-1.7440; -0.3731]	**0.0234**	0.0519 [-0.5211; 0.6249]	**$<10^{-4}$**	-0.3243 [-0.9107; 0.2621]	**$<10^{-4}$**
Black Africans and Caribbeans (34)	-0.0327 [-0.6720; 0.6067]		-0.5995 [-0.8191; -0.3799]		-0.5533 [-0.7716; -0.3349]	
North Africans and Middle East (37)	-0.3084 [-0.7550; 0.1383]		-0.1559 [-0.3841; 0.0723]		-0.1328 [-0.3602; 0.0946]	
Others (1)	NA	NA	-0.3684 [-1.2853; 0.5484]		-0.5901 [-1.6502; 0.4699]	

Main cause of end-stage renal disease (vs. hypertension, n= 62)						
Chronic interstitial nephropathy and pyelonephritis(24)	-0.3444 [-0.8360; 0.1473]	0.0897	-0.1434 [-0.4459; 0.1591]	0.6245		
Diabetes mellitus (39)	0.1120 [-0.4146; 0.6386]		-0.0380 [-0.3367; 0.2606]			
Dysimmune nephropathy including lupus and vascularitis (11)	-0.7609 [-1.6250; 0.1032]		-0.2533 [-0.6658; 0.1592]			
Glomerulopathy including IgA nephropathy (90)	0.0156 [-0.3890; 0.4202]		-0.2236 [-0.4490; 0.0018]			
Polycystic kidney disease (73)	0.1059 [-0.2943; 0.5062]		-0.1762 [-0.3946; 0.0422]			
Uropathy including reflux nephropathy (13)	-0.0596 [-0.6551; 0.5358]		-0.2653 [-0.6872; 0.1565]			
Undetermined (68)	-0.4004 [-0.7932; -0.0075]		-0.2133 [-0.4332; 0.0067]			
Other (14)	-0.3025 [-0.9580; 0.3529]		-0.0409 [-0.4223; 0.3405]			
Cardiovascular disease (Y/N)	-0.0949 [-0.3953; 0.2054]	0.5379	0.1749 [0.0047; 0.3452]	0.0453		
Diabetes at baseline	0.1889 [-0.2107; 0.5885]	0.3577	0.0397 [-0.1734; 0.2529]	0.7152	0.1281 [-0.0466; 0.3027]	0.1519
Dyslipidaemia at baseline	0.3939 [0.1263; 0.6614]	**0.0053**	0.0355 [-0.1050; 0.1761]	0.6209	0.0879 [-0.0590; 0.2349]	0.2419
BMI at D0	0.0013 [-0.0301; 0.0327]	0.9350				
Requirement for dialysis over the 1st week post-transplant (Y/N)	-0.0073 [-0.4223; 0.4077]	0.9727				
Number of dialyses	-0.0109 [-0.0946; 0.0728]	0.7993				
Corticosteroids (Y/N)	NA	NA	-0.0994 [-0.2631; 0.0642]	0.2350		

Laboratory test results over the targeted periods						
Total bilirubin	0.0371<br>[-0.0093; 0.0835]	0.1217				
ASAT					-0.0001<br>[-0.0003; 0.0002]	0.6773
Gamma GT	-0.0001<br>[-0.0012; 0.0011]	0.9206				
Haematocrit	0.0385<br>[0.0115; 0.0655]	**0.0065**				
Plasma creatinine	0.0008<br>[-0.0001; 0.0017]	0.0927				
eGFR	0.0009<br>[-0.0032; 0.0050]	0.6777				
Urine creatinine	0.0023<br>[-0.0269; 0.0316]	0.8760				
Number of C0/Dose	0.0940<br>[-0.0269; 0.2148]	0.1324				
Timing of tacrolimus intake	NA	NA	0.0178<br>[-0.2053; 0.2408]	0.8761		

**Patient numbers in brackets are for the period D0-D7.*

NA: not assessed

Cells are left empty when variables were not significant at the univariate stage.

Table S3: Multilinear analysis of intra-individual variability factors of tacrolimus log(C_0/D) over M3-M12 (n=254).

**Variables selected by univariate analysis**	**Beta [2.5%; 97.5%]**	**P value**
Chronic hepatic disorder	-1.301 [-2.484; -0.1185]	0.01964
Cancer	-1.347 [-2.409; -0.2842]	0.01319

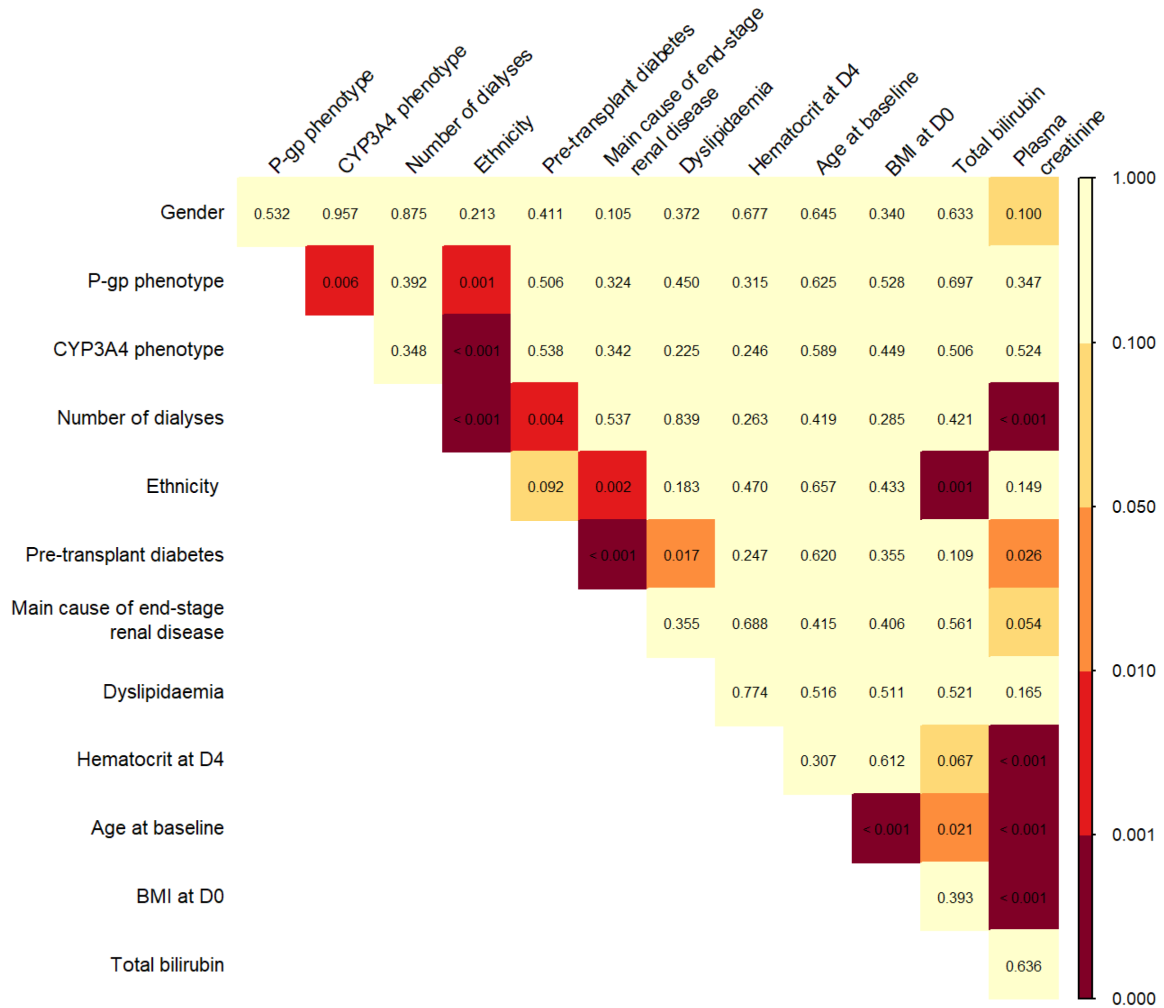

Figure S1. Heatmap of the p-values between relevant variables tested in pairs in the FAS population. Pairs of qualitative variables were tested using the Chi2 test. Pairs of quantitative variables using the Spearman test, and mixed pairs using the Kruskal-Wallis test.

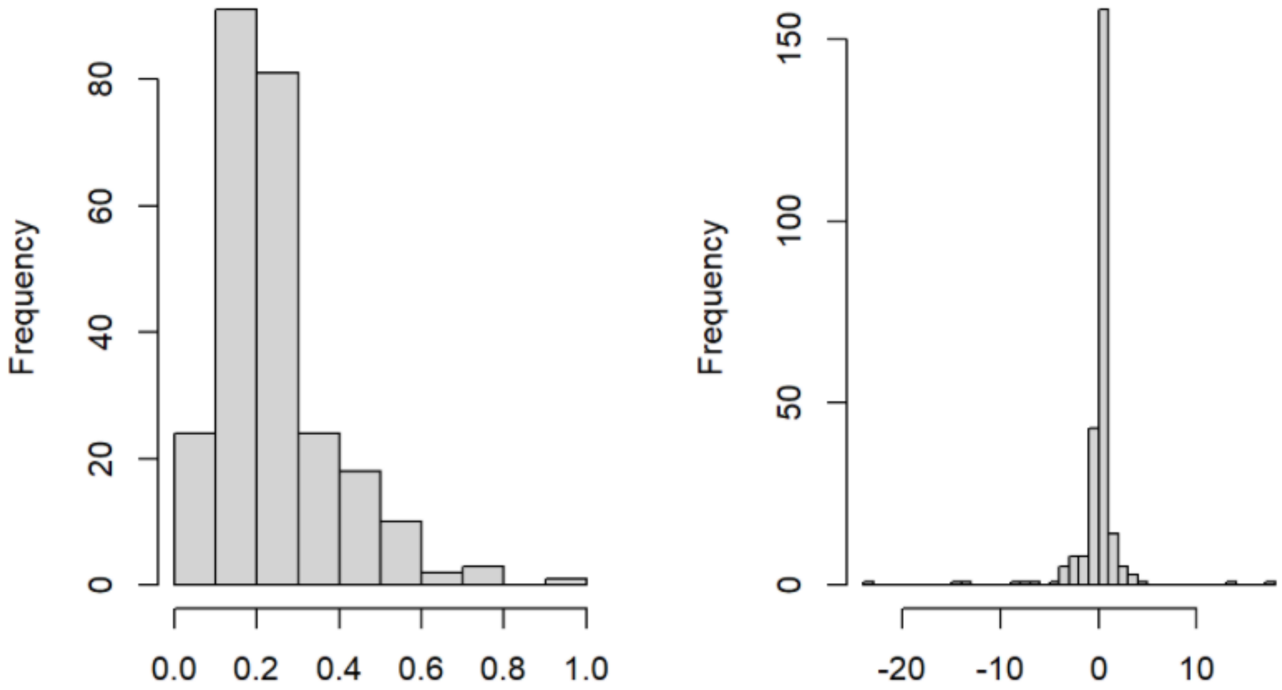

Figure S2. Inter-occasion variability: intra-patient coefficient of variation (CV%) of C0/D and log(C0/D) in 254 patients with at least 3 C0 values saved in the eCRF over the M3-M12 period.
